# Sacubitril/Valsartan and Right Ventricular and Diastolic Function During Breast Cancer Therapy

**DOI:** 10.1016/j.jaccao.2026.06.002

**Published:** 2026-08-18

**Authors:** Mari Nordbø Gynnild, Albulena Mecinaj Lilleaasen, Torgeir Wethal, Even O. Jakobsen, Victoria Vinje-Jakobsen, Siri Lagethon Heck, Stian Larsen, Assami Rösner, Havard Dalen, Torbjørn Omland, Geeta Gulati, Espen Holte

**Affiliations:** a K.G. Jebsen Center for Cardiac Biomarkers, Institute of Clinical Medicine, University of Oslo, Norway; b Department of Cardiology and Cardiothoracic Surgery, St Olavs University Hospital, Trondheim, Norway; c Department of Circulation and Medical Imaging, Faculty of Medicine and Health Science, NTNU – Norwegian University of Science and Technology, Trondheim, Norway; d Department of Cardiology, Division of Medicine, Akershus University Hospital, Norway; e Stroke Unit, Clinic of Medicine, St Olavs University Hospital, Trondheim, Norway; f Department of Neuromedicine and Movement Science, Faculty of Medicine and Health Science, NTNU – Norwegian University of Science and Technology, Trondheim, Norway; g Department of Diagnostic Imaging, Akershus University, Hospital, Lørenskog, Norway; h Department of Cardiology, Stavanger University Hospital, Stavanger, Norway; i Department of Cardiology, Division of Cardiothoracic and Respiratory Medicine, University Hospital of North Norway, Tromsø, Norway; j Department of Clinical Medicine, UiT The Arctic University of Norway, Tromsø, Norway; k Department of Medicine, Levanger Hospital, Nord-Trøndelag Hospital Trust, Levanger, Norway; l Department of Cardiology, Division of Medicine, Oslo University Hospital, Ullevål, Norway

**Keywords:** anthracycline, breast cancer, echocardiography, heart failure, prevention

**Figure:**
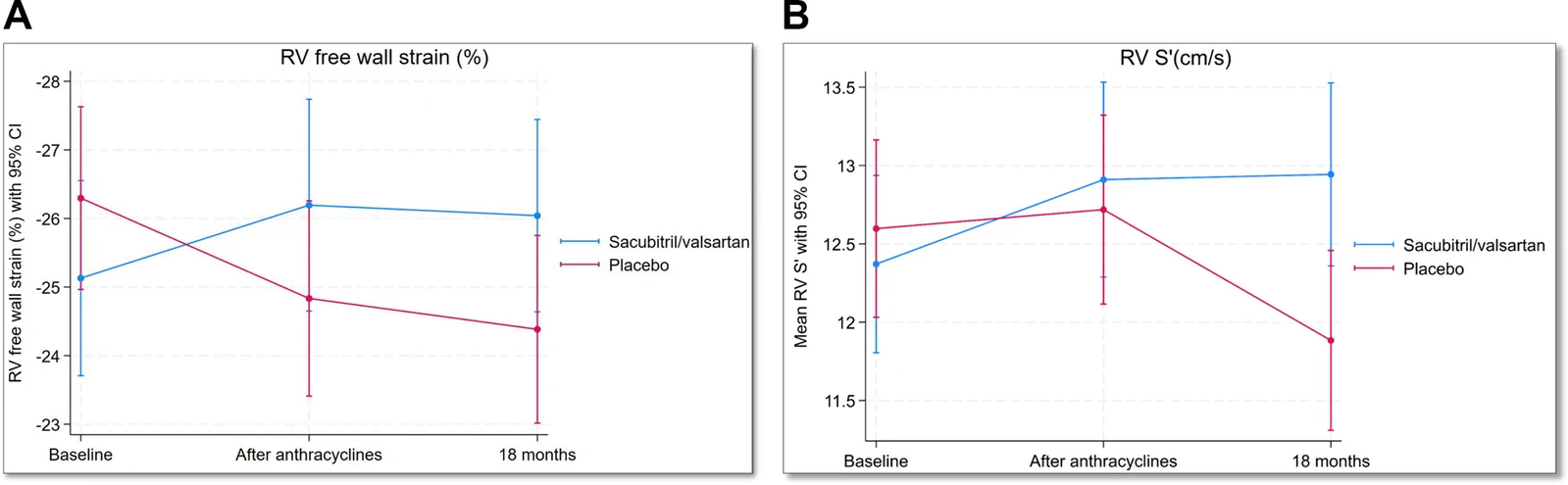

Anthracycline-based chemotherapy remains integral to early breast cancer treatment but carries risk of myocardial injury that may progress to heart failure.^1^ Beyond left ventricular (LV) systolic function, right ventricular (RV) systolic function and LV diastolic indices may provide earlier markers of subclinical cardiac dysfunction. The angiotensin receptor–neprilysin inhibitor sacubitril/valsartan moderately attenuated increases in N-terminal pro–B-type natriuretic peptide and cardiac troponin I and preserved global longitudinal strain in the PRADA II (Prevention of Cardiac Dysfunction During Adjuvant Breast Cancer Therapy; NCT03760588) trial;^2^ however, the effects on RV systolic function and LV diastolic function in this setting have not been reported. We present a predefined analysis of these endpoints from the PRADA II trial.

PRADA II was a randomized, double-blind, placebo-controlled multicenter trial in which 138 women receiving adjuvant anthracycline-based chemotherapy (±trastuzumab) for breast cancer were randomized to sacubitril/valsartan (target dose 97/103 mg twice daily) or placebo, initiated before the first anthracycline dose and continued for 18 months.^2^ Echocardiography was performed at baseline (T0), after anthracycline completion (T1), and at 18 months (T2), and analyzed blinded to treatment allocation and study order at a European Association of Cardiovascular Imaging–accredited core laboratory.

RV systolic parameters included RV free wall longitudinal strain (FWLS), tricuspid annular plane systolic excursion (TAPSE), RV systolic velocity (S′), and RV fractional area change (FAC). Diastolic parameters included mitral e’ velocity, E/e′ ratio, E/A ratio, left atrial volume index, and tricuspid regurgitation velocity. Between-group differences in change from T0 to T2 were estimated using linear mixed-effects models with fixed effects for treatment group, visit (T0, T1, T2), and treatment × visit interaction, adjusted for study site and trastuzumab use, with a random intercept per participant. Results are presented as least squares mean differences with 95% CI. PRADA II was approved by the Regional Committees for Medical and Health Research Ethics (2017/2411) and all participants provided written informed consent.

Mean ± SD age was 54 ± 9.4 years; baseline cardiac function was preserved in both groups, with no patients meeting criteria for diastolic dysfunction and RV indices were within normal limits (RV S′ 12.5 ± 2.3 cm/s, RV FWLS −26.0% ± 4.3%). LV ejection fraction (2-dimensional biplane) declined modestly and similarly in both groups (ΔΔ −0.31%; 95% CI: −2.7 to 2.1), with no significant between-group difference. RV systolic function remained preserved throughout follow-up. RV S′ increased in the sacubitril/valsartan group (+0.6 cm/s) but declined with placebo (−0.7 cm/s), yielding a between-group difference of +1.28 cm/s (95% CI: 0.43-2.13; *P* = 0.003). RV FWLS improved modestly with sacubitril/valsartan (−0.9%) but deteriorated with placebo (+1.9%), with a between-group difference of −2.82% (95% CI: –5.21 to −0.44; *P* = 0.021). TAPSE and FAC showed no significant between-group differences. RV indices showed minimal correlation with systemic load variables, suggesting the RV effects are less likely to be explained by afterload reduction alone. Temporal trajectories for RV function are shown in Figure 1.

**Figure 1:**
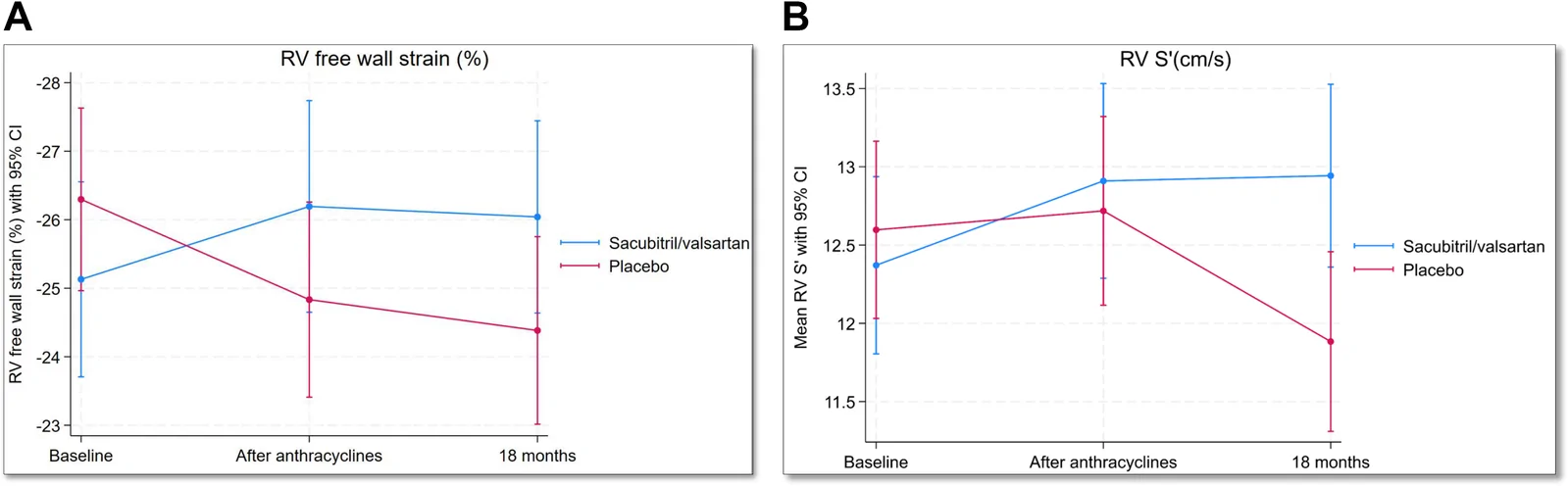
Temporal Changes in RV Systolic Function Estimates from mixed-model linear regression showing least squares mean (±95% CIs) trajectories of right ventricular (RV) free wall strain (A) and right ventricular systolic velocity (RV S′) (B).

Diastolic indices were within the normal range at baseline and changed modestly in both groups. Mitral e′ velocity was slightly higher with sacubitril/valsartan at follow-up (ΔΔ 0.83 cm/s; 95% CI: 0.12-1.53; *P* = 0.018). E/A ratio declined in the placebo group but remained stable with sacubitril/valsartan (ΔΔ 0.11; 95% CI: 0.00-0.21; *P* = 0.040). No significant between-group difference was observed for E/e′ ratio (ΔΔ −0.37; 95% CI: −0.99 to 0.25; *P* = 0.241). No between-group differences were observed for left atrial volume index or tricuspid regurgitation velocity. No patients met criteria for diastolic dysfunction during follow-up. Applying the Benjamini-Hochberg false discovery rate procedure across prespecified outcomes, only RV S′ remained statistically significant; remaining findings with *P* values between 0.01 and 0.05 should be considered hypothesis-generating.

In this randomized, placebo-controlled echocardiographic substudy of PRADA II, sacubitril/valsartan was associated with more favorable trajectories in sensitive RV systolic measures, specifically RV S′, with directionally consistent but hypothesis-generating trends for RV free wall strain. A similar directionally consistent trend was observed for mitral e’ velocity. LV ejection fraction and conventional RV indices (TAPSE, FAC) did not differ significantly between groups. These findings complement the previously reported effects on global longitudinal strain and cardiac biomarkers from the main PRADA II publication,^2^ and extend the literature by demonstrating RV-specific signal, which to our knowledge, has not previously been reported in a sacubitril/valsartan cardio-oncology trial.^3^, ^4^, ^5^ The clinical relevance of these modest findings in a predominantly low-risk population remains uncertain and should be considered hypothesis-generating. Whether attenuation of subclinical RV and diastolic changes translates into clinical benefit requires confirmation in larger studies enrolling higher-risk populations.

## Funding Support and Author Disclosures

This work was supported by The South-Eastern Norway Regional Health Authority, The University of Oslo, The Extra Foundation for Health and Rehabilitation, Norway, The Norwegian Cancer Society, and Akershus University Hospital. Study medications and matching placebos were provided free of charge by Novartis. The authors have no potential conflicts of interest with respect to the research, authorship, and/or publication of this article. Professor Omland received honoraria from Abbott Diagnostics, Roche Diagnostics, SpinChip, and Novo Nordisk and has received research support from Abbott Diagnostics, Novartis, ChromaDex, and Roche Diagnostics via Akershus University Hospital. Dr Gulati has received speaker honoraria from Astellas, Bayer, Beonemed, Bonnier Healthcare, NordicPharma, and Ipsen. All other authors have reported that they have no relationships relevant to the contents of this paper to disclose.
